# Athletic Identity and Shoulder Overuse Injury in Competitive Adolescent Tennis Players: The Smash Cohort Study

**DOI:** 10.3389/fspor.2022.940934

**Published:** 2022-07-06

**Authors:** Fred Johansson, Ulrika Tranaeus, Martin Asker, Eva Skillgate, Fredrik Johansson

**Affiliations:** ^1^Department of Health Promotion Science, Musculoskeletal and Sports Injury Epidemiology Center, Sophiahemmet University, Stockholm, Sweden; ^2^Unit of Intervention and Implementation Research for Worker Health, Institute of Environmental Medicine, Karolinska Institutet, Stockholm, Sweden; ^3^The Swedish School of Sport and Health Sciences, Gymnastikoch Idrottshögskolan (GIH), Stockholm, Sweden; ^4^Scandinavian College of Naprapathic Manual Medicine, Stockholm, Sweden

**Keywords:** tennis, injury, overuse injury, athletic identity, shoulder

## Abstract

**Objectives:**

Our primary aim was to determine if athletic identity is prospectively associated with shoulder overuse injuries. Secondly, we aimed to determine if athletic identity is prospectively associated with playing through pain and to describe how athletic identity relates to sex, age, playing level, weekly training load, and match volume.

**Methods:**

A cohort of 269 adolescent tennis players were followed over a period of 52 weeks. Cox regression was used to estimate the hazard rate ratio (HRR) of first-time shoulder overuse injury associated with every 10-unit increase on the Athletic Identity Measurement Scale (AIMS).

**Results:**

The adjusted HRR of shoulder overuse injury was 0.89 (95% CI: 0.36–2.20) and the odds ratio of playing through pain was 2.41 (95% CI: 0.74–8.96) for every 10 unit increase on AIMS. The level of athletic identity was higher among players at the national level than among players at the regional level and was weakly correlated to weekly hours of tennis matches, tennis training, and fitness training.

**Conclusions:**

Our results indicate that higher levels of athletic identity may be associated with a lower incidence of shoulder overuse injuries, and potentially with playing through pain, although these results are inconclusive due to wide confidence intervals.

## Introduction

Tennis is one of the most physically challenging individual sports requiring high levels of muscular strength, speed, power, agility, mobility, aerobic fitness, and anaerobic power output during matches in some competitions (König et al., 2001). However, tennis is not only about physiology but also the psychological factors become crucial already at a young age due to its competitive structure and the rule of no coaching during matches (De Muynck et al., 2020). In addition, injuries are common in elite adolescent tennis players, where injury rates of 1.2–2.8 injuries per 1,000 h played have been reported (Pluim et al., 2016; Gescheit et al., 2019; Moreno-Perez et al., 2019) with an overall yearly injury prevalence of 41% (Kovacs et al., 2014). Moreover, once the first injury has occurred, the likelihood of a second injury is as high as 31% (Kovacs et al., 2014). Overuse injuries are particularly troublesome, as they are the most frequently reported health complaint among adolescent tennis players, with a weekly prevalence of 12.1% (Pluim et al., 2016).

Shoulder overuse injuries pose a substantial problem for athletes with repetitive overhead arm motions, and they are likely to be multifactorial (Bahr and Krosshaug, 2005). Although the evidence is limited and further research is needed, a variety of risk factors of shoulder injuries in overhead sports have been suggested, such as decreased strength, scapular dyskinesia, and increased workload (Møller et al., 2017; Asker et al., 2018, 2020). Besides physiological and biomechanical factors (Kekelekis et al., 2020), psychological factors may further affect the risk of injury (Ivarsson et al., 2017). Psychological factors have been identified as an important focus in sports injury research (Johnson and Ivarsson, 2017), with athletic identity as one of the suggested factors (Visek et al., 2008).

Athletic identity refers to the degree to which a person identifies him- or herself as an athlete (Brewer et al., 1993). When the concept of athletic identity was introduced in the early 90's it was speculated that high levels of athletic identity could have both positive and negative effects for athletes (Brewer et al., 1993). It was hypothesized that while a salient athletic identity may increase the efforts invested by the players, it may also increase the risk of excessive training or competitive effort (i.e., playing while being injured; Brewer et al., 1993). Later research has confirmed athletic identity as an important motivational resource for athletes to prioritize sports and invest efforts in training (Stambulova et al., 2015). High levels of athletic identity are also associated with higher commitment to sports participation, better athletic achievement (Horton and Mack, 2000), and higher competitiveness (Daniels et al., 2005).

On the negative side, high levels of athletic identity show moderate positive associations with psychological distress following injury (Brewer, 1993; Manuel et al., 2002) and retirement from sports (Giannone et al., 2017). Further, high levels of athletic identity are moderately associated with higher behavioral inclinations to train and compete with pain or injury (Weinberg et al., 2013; Renton et al., 2021), and floorball players have been reported to ignore physical symptoms to protect their athletic identity (Tranaeus et al., 2014). These potentially risky behaviors can result from the norm and culture of acceptance of pain in sports (Roderick et al., 2000; Tranaeus et al., 2014), and may potentially link higher levels of athletic identity to higher rates of overuse injuries.

A recent explorative study using latent profile analysis, however, found no clear association between athletic identity and injury frequency among competitive athletes in various sports (Martin et al., 2021). To our knowledge, there is only one hypothesis-testing prospective study investigating the association between athletic identity and injury incidence. This study found that high levels of athletic identity were associated with a lower risk for the first injury, but a higher risk of repeated injuries among adolescent ice-hockey players (McKay et al., 2013).

Given the sparsity of prospective studies on this topic, we believe there is a substantial research gap concerning the prospective association between athletic identity and the incidence of overuse injuries, especially for athletes in individual sports.

Our primary aim was to determine if athletic identity is associated with shoulder overuse injuries in adolescent competitive tennis players. Secondly, we aimed to determine if athletic identity is prospectively associated with playing through pain. Thirdly, we aimed to examine how athletic identity relates to sex, age, playing-levels, and the volume of weekly training and matches. Our hypotheses were that a higher athletic identity would be related to a higher incidence of shoulder overuse injury and a higher likelihood of playing through pain. Thirdly, we hypothesized that athletic identity would be positively associated with playing level and volume of training and matches. We had no directed hypotheses regarding sex and age.

## Methods and Materials

### Participants and Procedures

The SMASH cohort study was conducted from February 2018 until March 2019. It included 301 adolescent competitive Swedish male and female tennis players (aged 12–19 years). Recently, two studies have been published on workload as a risk factor for shoulder overuse injuries and back pain, respectively (Johansson et al., 2022a,b). Our present investigation used longitudinal data from the SMASH cohort study to further deepen the knowledge about risk factors.

**Figure 1:**
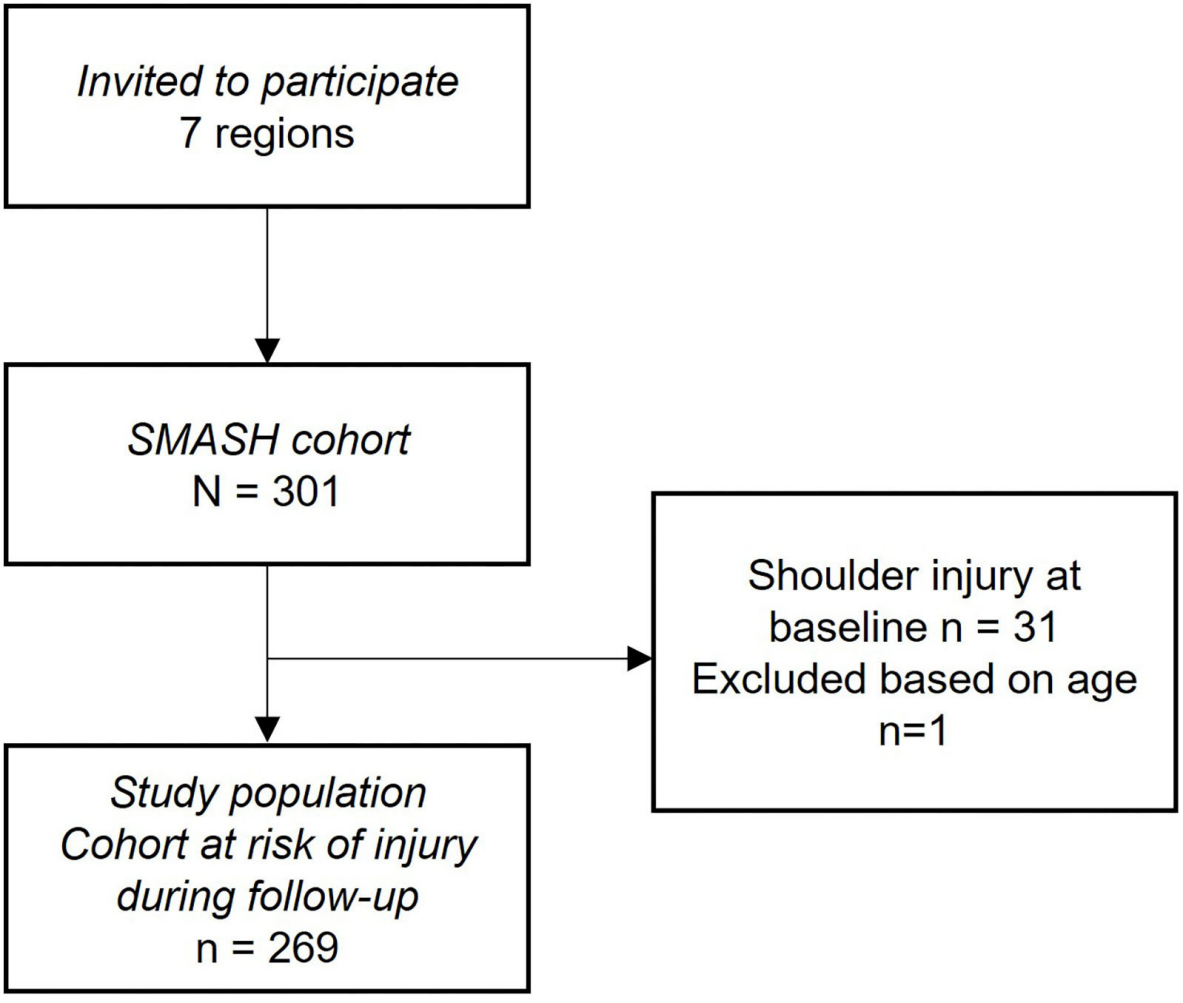
Flowchart describing the inclusion process.

Players from the Swedish Tennis Association regional and high-performance programs were invited to participate in the study and all invited players agreed to do so, representing all seven tennis regions in Sweden. The players were screened clinically (this information is not presented in this article) and filled out a comprehensive self-report questionnaire at baseline. Inclusion criteria for the SMASH-cohort were (1): competitive level of at least regional level in Sweden; (2): minimum of 8 h of total training volume per week on average. Players were excluded if they had had shoulder surgery or dislocation during the 6 months prior to baseline assessment. The included participants were followed for 52 weeks *via* a web-based questionnaire sent out every Sunday, with reminders sent out 24 h later if the participant had not responded. In the current study, participants were excluded if they reported a shoulder overuse injury during the last 3 months. Also, one participant falsely included on age was excluded (Figure 1).

Informed consent was provided by all participants. For participants under 15 years of age, the legal guardian of the participant gave their written informed consent. The study was conducted in accordance with the declaration of Helsinki and approved by the Regional Ethical Review Board, Stockholm, Sweden (approval no. 2012/1731/2).

### Measures

Descriptive information, such as age, sex, and years of playing tennis, was assessed in the baseline self-report questionnaire. The level of play was classified as regional or national level with support from the Swedish Tennis Association high-performance program. Players were followed weekly regarding external workload (How many hours and minutes match play have you performed the preceding week? How many hours and minutes have you practiced tennis on the tennis court the preceding week?).

Shoulder overuse injuries were measured weekly using the Oslo Sports Trauma Research Centre Overuse Injury Questionnaire (OSTRC-O; Clarsen et al., 2013; Ekman et al., 2015). The questionnaire includes four items covering (1): difficulties to participate in sports; (2): reductions in training volume; (3): affected performance, and (4): shoulder-related pain. Each item is scored from 0 to 25 giving a total score ranging from 0 to 100, with overuse injury defined as a total score ≤ 40 (Clarsen et al., 2014).

Playing through pain was measured weekly and defined based on two of the OSTRC-O questions (Clarsen et al., 2013): “Have you had difficulties participating in sports (training, matches, and competitions) due to shoulder problems during the last week?” and “To what extent have you experienced shoulder pain related to your sport during the last week?” The answer “Participated fully with shoulder problems” in combination with a moderate or severe pain rating was classified as playing through pain. Players reporting at least 1 week of playing through pain were classified as having played through pain and compared to players reporting no weeks of playing through pain.

The Athletic Identity Measurement Scale seven item version *(AIMS)* was administered at baseline and used to measure the level of athletic identity (Visek et al., 2008). This self-rated scale consists of seven items scored on a 5-point Likert-scale ranging from 1 (strongly disagree) to 5 (strongly agree). Originally, the scale has a 7-point Likert-scale but this was changed to a 5-point scale in our Swedish version, with a total score ranging from 5 to 35. The Cronbach's alpha of AIMS was 0.62 in our sample.

### Confounders

Potential confounders were identified from previous literature and drawn in a directed acyclical graph (Lederer et al., 2019) in which sex and playing level (regional or national level) were identified as potential confounders (see Supplementary Figure 1).

### Statistical Analyses

Demographic data and descriptive data, such as training background, average weekly training volume, and level of play are summarized in Table 1 as mean levels or proportions.

**Table 1:** Sample characteristics.

	**Full sample** **(*n* = 269)**
Sex, *n* (%)	
Female	114 (42%)
Male	155 (58%)
Age, M (SD)	14.5 (2.0)
Years of playing tennis, M (SD)	8.5 (2.6)
Playing level, *n* (%)	
National	45 (17%)
Regional	224 (83%)
Ever experienced shoulder pain while playing tennis, n (%)	143 (53%)
Weekly match hours^†^, M (SD)	2.5 (2.1)
Weekly hours of tennis practice^†^, M (SD)	7.4 (3.1)
Weekly hours of fitness training^†^, M (SD)	2.6 (1.5)
Participating in other sports, n (%)	83 (31%)

*All measures were collected at baseline except those marked with ^†^ which are weekly average until first injury or censoring*.

**Figure 2:**
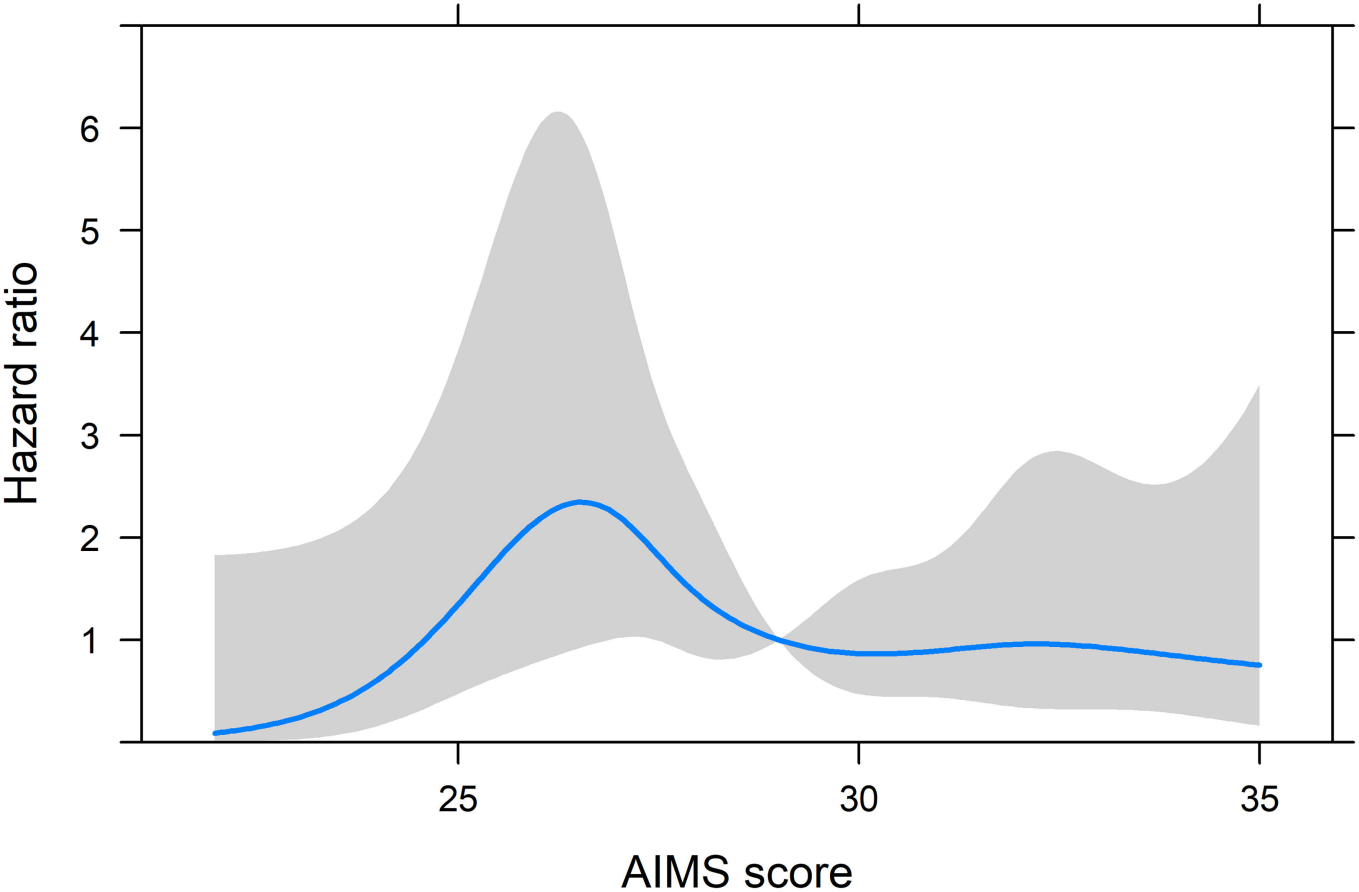
Hazard rate ratio of shoulder overuse injury by AIMS score from the *post-hoc* model with restricted cubic splines on the AIMS variable and the mean as the reference point. The shaded area represents the 95% CI. Adjusted to: Playing level, Regional; Sex, Male.

A multivariate Cox proportional hazard model was built to assess the change in hazard rate ratio (HRR) of shoulder overuse injury for every 10 unit increase on AIMS and presented with 95% confidence intervals (CIs). AIMS was treated as a continuous variable and its coefficient was transformed to reflect 10-unit increases for interpretability. The model was adjusted for playing level (regional or national) and sex. The assumption of proportional hazards was tested by plotting the scaled Schoenfeld residuals against time to event or censoring. Linearity was assumed *a priori* in the model above given the first hypothesis. The validity of the assumption of linearity was assessed by plotting the martingale residuals against the AIMS variable. Non-linearities in the association between AIMS and incidence of overuse injury were explored *post-hoc* using restricted cubic splines (Harrell, 2015). A Cox proportional hazards model with knots at the 5th, 25th, 50th, 75th, and 95th percentile of the AIMS variable was fitted and adjusted for the covariates described above. The HRR across the range of AIMS is presented graphically in Figure 2 along with 95% CI:s, with the mean AIMS score as the reference category. The evidence for an association between the AIMS score and overuse injury in this model was evaluated by performing a Wald's *X*^2^ test on all coefficients related to AIMS (Harrell, 2015). Time at risk was defined as the total number of hours of tennis exercise and match-play until injury or censoring. Missing observations led to that week being omitted from the time at risk summation, but also these players could not report injuries during these weeks. Players were censored when an injury was reported or if no injury was reported at the end of the study. Players who contributed no weekly follow-ups did not contribute to the risk analysis (*n* = 24).

The odds ratio (OR) of playing through pain at any time during the 52 weeks (no censoring) associated with 10-unit increases on AIMS was calculated using logistic regression and presented with 95% CI. As with the Cox model above, this model was adjusted for sex and playing level.

Differences in levels of athletic identity between sexes and the playing levels were calculated as mean differences and presented along with 95% CIs from *t*-tests. Associations between AIMS score and age, weekly training and match volume, and shoulder strength were calculated as Pearson's correlations and presented along with 95% CIs.

No *a priori* sample size calculations were made relating to this study, as the research questions addressed here were not the primary research questions of the SMASH cohort study data collection.

All analyses were performed in RStudio version 1.2.5001. Cox regressions were performed using the “survival” and “survminer” packages. The restricted cubic splines Cox model was fitted using the “rms” package (Harrell, 2021).

## Results

Out of the total 301 players included in the SMASH-cohort, 269 players were included in our analyses. The sample consisted of 42% women and the mean age was 14.5 years (SD 2.0; Table 1). The weekly response rate was 85% in the full cohort. Over the course of 52 weeks, 44 players reported shoulder overuse injuries.

### Risk Analysis

The crude hazard rate ratio (HRR) of shoulder overuse injury in the main analysis was 0.72 (95% CI: 0.30–1.69) for every 10-unit increase on AIMS, while the adjusted HRR was 0.89 (95% CI: 0.36–2.20). Since a visual inspection of the Martingale residuals indicated non-linearities, a *post-hoc* analysis using a restricted cubic splines model was performed (Figure 2). Visual inspection of Figure 2 showed the highest HRR for intermediate levels of AIMS with lower HRR for higher and lower scores. However, the confidence intervals were wide (Figure 2), and the Wald's *X*^2^ test of the AIMS coefficients indicated a non-significant relationship between AIMS and shoulder overuse injuries (*p* = 0.19).

### Playing Through Pain

Over the 52 weeks, 28 players met the definition of playing through pain at any time. The crude OR of playing through pain was 1.87 (95% CI: 0.61–6.41) for every 10-unit increase on AIMS. The adjusted OR was 2.41 (95% CI: 0.74–8.96).

### AIMS Score and Demographics, Playing Level, and Training Volume

The mean AIMS score was 29.3 among women and 28.6 among men [mean difference 0.7 (95% CI: −0.1 to 1.6)]. The correlation between AIMS score and age was *r* = −0.2 (95% CI: −0.3 to −0.0). Players at the national level had a mean AIMS score of 31.1, while players at the regional level had a mean score of 28.4 [mean difference was 2.7 (95% CI: 1.6–3.8)]. AIMS scores correlated to average weekly match hours *r* = 0.2 (95% CI: 0.1–0.3), average weekly tennis practice *r* = 0.2 (95% CI: 0.1–0.3), and weekly hours of fitness training *r* = 0.1 (95% CI: 0.0–0.3).

## Discussion

The primary aim of this study was to explore the relationship between athletic identity and shoulder overuse injury in adolescent competitive tennis players. Contrary to our first hypothesis, there was a tendency toward decreased injury incidence for players with higher AIMS scores, although this trend is uncertain due to wide confidence intervals. Following the main analysis, we conducted a *post-hoc* analysis with restricted cubic splines on the AIMS score variable, allowing for non-linearities in the association with injury incidence. The results in Figure 2 again showed a tendency toward a lower risk of overuse injuries for players with high as well as low AIMS scores and an increased risk with intermediate levels of AIMS scores. These estimates should also be interpreted with caution since the Wald's *X*^2^ test did not show a significant overall effect of AIMS score on HRR of shoulder overuse injury. Although our results are inconclusive, they are in line with previous findings, showing that young ice-hockey players with high levels of athletic identity had a lower risk for first injury (McKay et al., 2013). The results are however at odds with research and theories, suggesting an increased risk of overuse injury in players with high levels of athletic identity (Brewer et al., 1993; Tranaeus et al., 2014). It has been argued that single risk factor models, like in the present analysis, may overlook the complexity of the multi-casual etiology of sports injuries (Bittencourt et al., 2016). It is possible that factors omitted in our analysis may interact with athletic identity affecting injuries, something we believe needs attention in future research.

Athletic identity was hypothesized to be positively associated with playing through pain, which was one mechanism believed to potentially link high levels of athletic identity with overuse injury (Supplementary Figure 1). Our results showed a tendency toward a higher likelihood of playing through pain for players with a higher level of athletic identity, but the wide confidence interval leaves the estimate uncertain. This tendency is in line with earlier research on inclinations to play through pain among players with high levels of athletic identity (Visek et al., 2008; Renton et al., 2021). It may seem paradoxical that players with high athletic identity could potentially play more with pain, but still contract fewer overuse injuries. As discussed earlier, this could be since athletic identity may also be associated with protective factors decreasing the risk of overuse injuries.

We further found that athletic identity was slightly higher among players at the national level than among players at the regional level. Athletic identity also correlated positively to weekly hours of tennis matches, tennis training, and fitness training. There were no clear associations between sex or age and athletic identity. Overall, these results indicate that athletic identity is associated with playing at a higher level and investing more time and effort in training. Moreover, the results are in line with findings from other sports suggesting that high levels of athletic identity may be related to higher achievement and investing more effort into one's sport (Horton and Mack, 2000; Stambulova et al., 2015). Although we cannot infer causality from these associations, it is possible that adolescent players at a higher competitive level may act and behave more professionally off-court, including preparation, fitness, and recovery, which may in part explain the trend toward reduced risk of shoulder overuse injury.

### Strengths and Limitations

Our study had several strengths but also limitations. First, this unique cohort of 269 adolescent competitive tennis players comprises nearly all players at this level in Sweden. Secondly, prospective data were collected for 52 consecutive weeks with a high weekly follow-up rate of 85%, limiting the risk of selection bias in the results. Thirdly, both AIMS and OSTRC-O are valid measures, which lower the risk of misclassification. However, classifying players as injured using the OSTRC-O relies partly on the reduction of participation during matches and training. This may be problematic in relation to the athletic identity as high levels of athletic identity have been hypothesized to lead to excessive training even when injured. Also, adolescence is a time of identity formation, and it is possible that athletic identity changed for some players during the study time, which would lead to misclassification over time and a dilution of the effects. Another limitation is that the internal consistency of the AIMS was questionable in this sample (Cronbach's α = 0.62). The novel usage of the OSTRC-O to measure playing through pain has not been validated. However, given the short recall time and the straightforwardness of the questions, we believe this provides a valid indicator of whether players have played with pain. Fourthly, we have considered several potential confounding factors when planning the analyses, which is a strength, even though the risk of unmeasured and residual confounding cannot be ruled out.

The main limitation is the lack of statistical power. Even though nearly all eligible players in Sweden were recruited and the effect measure for the risk analysis was rather strong, the estimates are too imprecise to draw any firm conclusions. The lack of power is mainly a result of the outcome being relatively uncommon in our sample, and 16% (*n* = 44) of the players experienced a shoulder overuse injury during the 1-year follow-up.

## Conclusion

Our results showed tendencies suggesting that higher levels of athletic identity may be associated with a lower incidence of shoulder overuse injuries in competitive adolescent tennis players, although the results are inconclusive due to wide confidence intervals. Playing through pain showed a tendency toward being more common among players with higher athletic identity; however these results should be interpreted with caution. Also, our results showed that athletic identity is positively related to playing at a higher level and spending more time training and playing matches, but not clearly associated with age or sex.

### Clinical Recommendations

In our view, the clinical implication of our study should be directed mostly toward coaches who are often the ones responsible for adolescent tennis players' training and competition calendars. Our results suggest that players with higher levels of athletic identity may not be at a higher risk of injury generally, although they showed a tendency toward playing through pain more often. Furthermore, the descriptive data on the variables in this study provide important benchmarking opportunities for coaches and clinicians serving competitive adolescent tennis players. Further studies and larger cohorts from many repetitive movement sports are needed to state this with more certainty.

## Data Availability Statement

The datasets analyzed for the current study are not publicly available due to secondary confidentiality and privacy of the participants. Requests to access the datasets should be directed to fredrik.johansson@shh.se.

## Ethics Statement

This study was approved by the Swedish Ethical Review Board, Sweden (approval no. 2012/1731/2). Written informed consent to participate in this study was provided by the participants' legal guardian/next of kin.

## Author Contributions

FredrJ and ES were responsible for the SMASH data collection with support from MA and UT. FredJ created the first draft of the manuscript and performed the statistical analyses. All authors were involved in formulating the research questions, analytic plan of the current study, and have contributed critical revisions to the intellectual content.

## Conflict of Interest

The authors declare that the research was conducted in the absence of any commercial or financial relationships that could be construed as a potential conflict of interest.

## Publisher's Note

All claims expressed in this article are solely those of the authors and do not necessarily represent those of their affiliated organizations, or those of the publisher, the editors and the reviewers. Any product that may be evaluated in this article, or claim that may be made by its manufacturer, is not guaranteed or endorsed by the publisher.

## Supplementary Material

Supplementary Figure 1 Directed acyclic graph of the assumed causal structure for our analyses.
