# Supplementary figures and images for: Athletic Identity and Shoulder Overuse Injury in Competitive Adolescent Tennis Players: The Smash Cohort Study
Source: Front Sports Act Living. 2022 Jul 6;4:940934. doi: 10.3389/fspor.2022.940934 (PMC9299246; doi:10.3389/fspor.2022.940934)

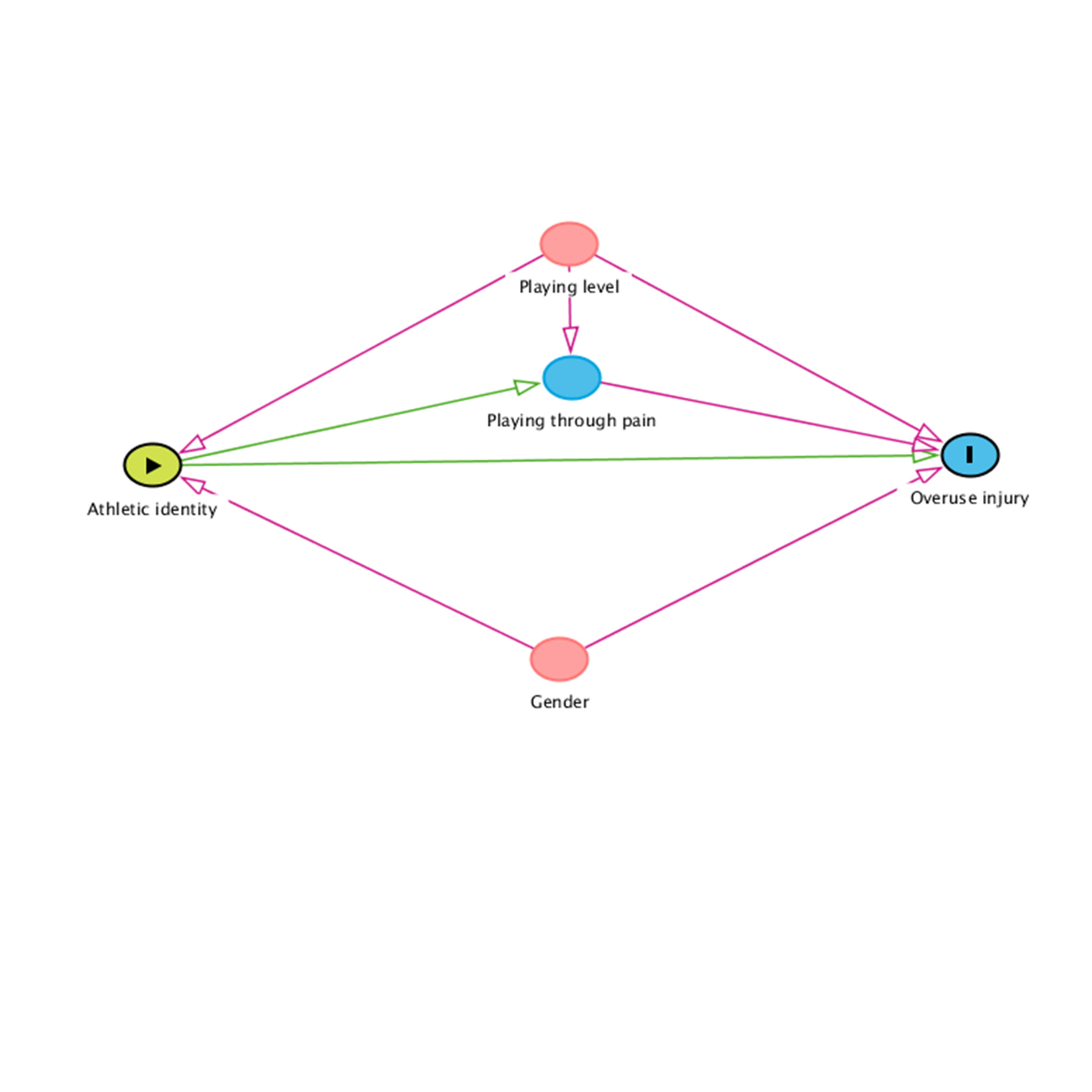

Supplement: Supplementary Figure 1 — Directed acyclic graph of the assumed causal structure for our analyses. [file Image_1.PNG]
